# Energy delivery guided by indirect calorimetry in critically ill patients: a systematic review and meta-analysis

**DOI:** 10.1186/s13054-021-03508-6

**Published:** 2021-02-27

**Authors:** Jing-Yi Duan, Wen-He Zheng, Hua Zhou, Yuan Xu, Hui-Bin Huang

**Affiliations:** 1 Department of Critical Care Medicine, Beijing Tsinghua Changgung Hospital, School of Clinical Medicine, Tsinghua University, Beijing, 102218 China; 2 Department of Critical Care Medicine, Rehabilitation Hospital affiliated to Fujian University of Traditional Chinese Medicine, Fuzhou, 350000 China

**Keywords:** Indirect calorimetry, Critically ill, Energy delivery, Mortality, Meta-analysis

## Abstract

**Background:**

The use of indirect calorimetry (IC) is increasing due to its precision in resting energy expenditure (REE) measurement in critically ill patients. Thus, we aimed to evaluate the clinical outcomes of an IC-guided nutrition therapy compared to predictive equations strategy in such a patient population.

**Methods:**

We searched PubMed, EMBASE, and Cochrane library databases up to October 25, 2020. Randomized controlled trials (RCTs) were included if they focused on energy delivery guided by either IC or predictive equations in critically ill adults. We used the Cochrane risk-of-bias tool to assess the quality of the included studies. Short-term mortality was the primary outcome. The meta-analysis was performed with the fixed-effect model or random-effect model according to the heterogeneity.

**Results:**

Eight RCTs with 991 adults met the inclusion criteria. The overall quality of the included studies was moderate. Significantly higher mean energy delivered per day was observed in the IC group, as well as percent delivered energy over REE targets, than the control group. IC-guided energy delivery significantly reduced short-term mortality compared with the control group (risk ratio = 0.77; 95% CI 0.60 to 0.98; *I*^2^ = 3%, *P* = 0.03). IC-guided strategy did not significantly prolong the duration of mechanical ventilation (mean difference [MD] = 0.61 days; 95% CI − 1.08 to 2.29; *P* = 0.48), length of stay in ICU (MD = 0.32 days; 95% CI − 2.51 to 3.16; *P* = 0.82) and hospital (MD = 0.30 days; 95% CI − 3.23 to 3.83; *P* = 0.87). Additionally, adverse events were similar between the two groups.

**Conclusions:**

This meta-analysis indicates that IC-guided energy delivery significantly reduces short-term mortality in critically ill patients. This finding encourages the use of IC-guided energy delivery during critical nutrition support. But more high-quality studies are still needed to confirm these findings.

**Supplementary Information:**

The online version contains supplementary material available at 10.1186/s13054-021-03508-6.

## Introduction

Determining energy requirements is the cornerstone of critical nutritional support. It has been demonstrated that avoiding underfeeding and overfeeding is an important factor directly related to nutritional support in critically ill patients [1]. Therefore, accurate and precise energy measurements in such a patient population are important. Various prediction equations are commonly practiced to predict resting energy expenditure (REE) currently [2]. However, these equations, which were originally developed basing data from healthy individuals, have been studied extensively and found to be mostly inaccurate in predicting REE in critically ill patients [2]. This is because these equations fail to consider the diversity of the ICU populations and the factors that influence REE [3]. Consequently, measured REE by some devices, such as indirect calorimetry (IC), has increasingly attracted attention.

IC is a validated, century-long studied technique that assesses REE by measuring the oxygen consumption and carbon dioxide production [4]. Some studies have recently evaluated the effects of IC-guided critical nutritional support in critical illness [5–7]. The tight calorie control study (TICACOS) compared IC-guided nutrition therapy to a single weight-based measurement and found an improvement in 60-day survival in the per-protocol study in the IC group (57.9% vs. 48.1%, *P* = 0.023) [8]. Results from further RCTs using IC as a energy target showed significant reduction in nosocomial infections [9] and improved immunity or less systemic inflammation [5]. Thus, the 2018 ESPEN guideline made a weak recommendation (grade B) favoring the use of IC to determine energy requirements in critically ill ventilated patients [10].

A published meta-analysis showed that compared with predictive equations, the IC-guided energy delivery strategy was not more effective in reducing hospital mortality, duration of MV, and length of stay (LOS) in ICU or hospital [11]. However, only four studies have been included. Thus, no firm conclusions can be drawn about the efficacy of IC-guided strategy in critically ill patients.

Recently, several studies on this topic have been published [12–15], and some of these studies have a modest sample size, while the conclusions are inconsistent. Therefore, with the aid of increased power of meta-analytic techniques, we sought to investigate whether IC-guided energy delivery strategy in critically ill patients may be more effective in reducing short-term mortality and other clinical outcomes than PEE predictive equations.

## Methods

We performed the current meta-analysis and systematic review according to the preferred reporting items for systematic reviews and meta-analyses (PRISMA) statement [16]. Our protocol was registered on the International Platform of Registered Systematic Review and Meta-analysis Protocols database (INPLASY2020110084) and is available in full on inplasy.com (https://doi.org/10.37766/inplasy2020.11.0084). Ethical approval was not required for this work.

### Search strategy and selection criteria

Two authors (J-YD and HZ) conducted a computerized search of PubMed, EMBASE, and the Cochrane Center Register of Controlled Trials (CENTRAL) from inception through October 25, 2020, which was the date of our last search. We used medical subject headings, keywords, and Emtree terms to search published RCTs that compared repeated REE measurements by IC to predicted equations measurements in guiding energy delivery in critically ill patients. The details in the search strategy are in Additional file 1. No language limitation was applied. We also reviewed the references listed of relative articles to identify potentially relevant studies.

### Selection criteria and outcomes

RCTs were considered for inclusion if they evaluated critical adult patients (≥ 18 years) receiving energy either guided by repeated IC (IC group) or a simple predictive equation (control group). Studies that used the combined energy delivery and any other nutritional regimens were excluded. We excluded studies recruiting children, breastfeeding women or pregnant, or studies without reporting any predefined outcomes. Animal studies, case reports, and reviews were excluded. Articles available only in abstract form or meeting reports were also excluded.

The primary outcome was short-term mortality (defined as ICU or hospital mortality or mortality within a 90-day follow-up after admission, with the longest observation period preferred [17]). Secondary outcomes included clinical nutrition parameters after treatment (i.e., REE targets, mean energy or protein delivered, defined by each author), duration of MV, length of stay (LOS) in ICU or hospital, long-term mortality (defined as mortality between hospital discharge and at least 180 days follow-up thereafter), and adverse events (AEs, defined by each study author).

### Data extraction and quality assessment

Two authors (J-YD and HZ) independently extracted data from included RCTs on study design, sample size, patient characteristics, IC measurements, and predictive equations measurements), and predefined outcomes. J-YD and HZ also independently evaluated potential evidence of bias using the Cochrane risk-of-bias tool for RCTs [18]. We assigned a value of high, unclear, low to the following items: (1) sequence generation; (2) allocation concealment; (3) blinding; (4) incomplete outcome data; (5) selective outcome reporting; and (6) other sources of bias. Discrepancies were identified and resolved by consensus or in discussion with a senior author (H-BH).

### Data analysis

The results were combined to estimate the pooled risk ratio (RR) and associated 95% confidence intervals (CI) for dichotomous outcomes. As to the continuous outcomes, mean differences (MD) and 95% CI were estimated. We calculated pooled estimates and proportions with 95% CI using the Freeman–Tukey double-arcsine transformation. Some studies reported median as the measure of treatment effect, with accompanying interquartile range (IQR). Before data analysis, we estimated mean from median and standard deviations (SD) from IQR [19]. Heterogeneity was tested by using the *I*^2^ statistic. An *I*^2^ < 50% was considered to indicate insignificant heterogeneity, and a fixed-effect model was used, whereas a random-effect model was used in cases of significant heterogeneity (*I*^2^ > 50%). Publication bias was assessed by visual inspection of funnel plots. All statistical analyses were performed using STAT Version 12.0 and Review Manager Version 5.3.

## Results

### Trial identification and characteristics

**Fig. 1:**
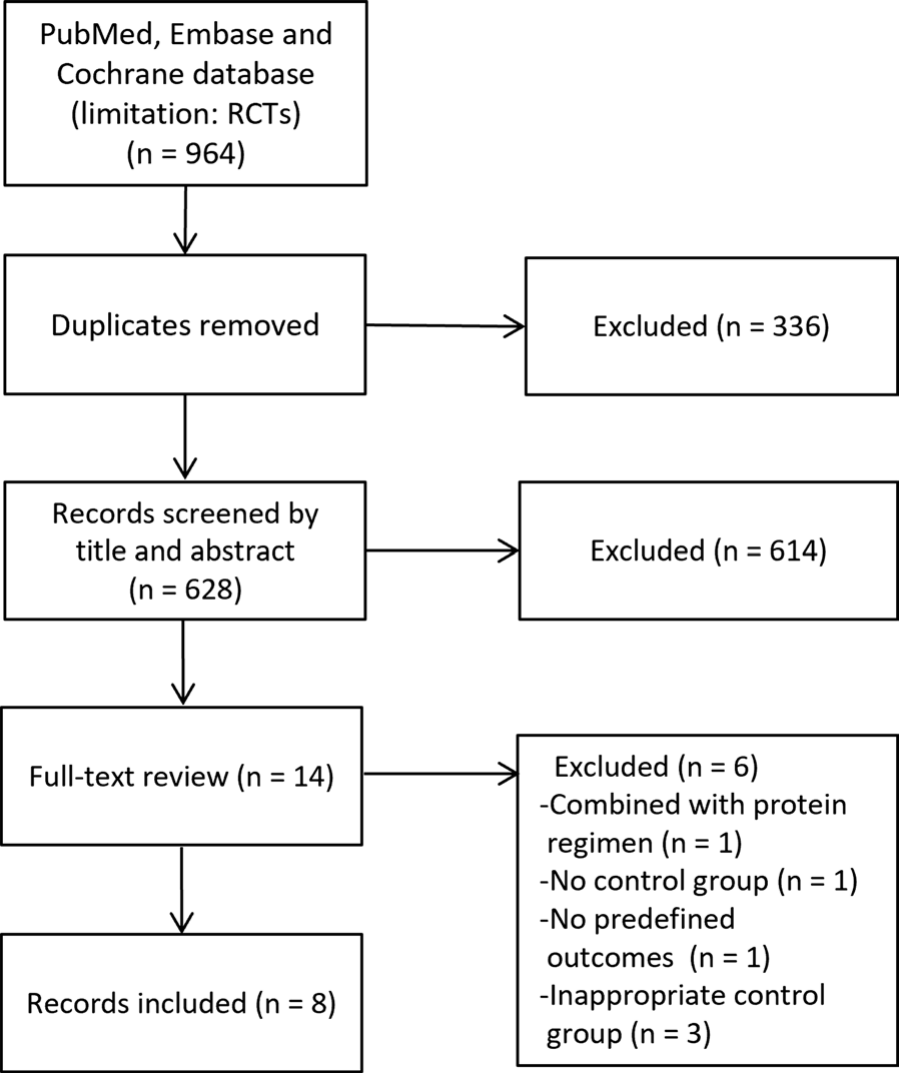
Selection process for studies included in the meta-analysis

Our literature search yielded 964 potentially eligible articles through database searching. Further screening of 14 full texts identified 8 RCTs with 911 patients that fulfilled our inclusion criteria and was included in the final analysis [7, 13–15, 20–23]. Figure 1 shows the flowchart of the search strategy and the reasons for exclusion. Table 1 presents the main characteristics of the included RCTs. The details of predefined outcomes are summarized in Additional file 2. These studies were published from 2011 to 2020. Seven out of the eight trials were single-center studies [7, 12, 15, 20–23]. All the eight RCTs recruited ventilated patients (sample size ranges from 40 to 417 cases), with 489 in the IC group and 484 in the control group, respectively. The IC measurements and types of IC devices used were described in all trials. As to the predictive equations used in the control group, a simplistic weight-based 25 kcal/kg per day and Harris–Benedict formula were used in 4 [7, 14, 20, 21] and 3 [13, 15, 23] RCTs, respectively, while both were used in another trial [22]. The quality of the included RCTs was moderate, with the high-performance bias related to the lack of blinding of participants and personnel among most studies (Additional file 3). No obvious publication bias was observed by a visual inspection of the funnel plots in the present meta-analysis (Additional file 4).

**Table 1:** Characteristics of the included studies in the current systemic review and meta-analysis

Study/year	Study design	Patient characteristics						Study regimen			
		Sample size	Age (years)	Male N%	BMI (kg/m^2^)	APACHE II	Population	Intervention		Nutrition therapy	Study period
		IC/PE	IC/PE	IC/PE	IC/PE	IC/PE		IC	PE		
Singer 2011 [7]	RC, SC	65/65	59/62	35/41	28/27	22/22	Ventilated patients, excepted to stay in ICU > 3 days	Measured every 2 days	25 kcal/kg/day	Aim of reaching energy goal within 24 h of inclusion (EN + PN)	Two weeks
Landes 2016 [22]	RC, SB, SC	15/12	72/74	60/83	25/26	35/39	Ventilated patients	Weekly test for 3 weeks	H–B formula or 25 kcal/kg/day	EN was guided by 110% IC in IC group and physician estimates in control group	Three weeks
Allingstrup 2017 [20]	RC, SB, SC	100/99	63/68	65/60	22/22	NR	Ventilated patients, excepted to stay in ICU > 3 days	Every other day	25 kcal/kg/day	IC: Aim to meet 100% EE in first 24 h (EN ± PN). Control: Increase in EN, supplemental PN if EE not met by Day 7	ICU discharge or Day 90
Gonzalez-Granda 2018 [21]	RC, SC	20/20	57/56	65/55	28/25	29/29	Ventilated patients, excepted to stay in ICU > 2 days	Weekly test	25 kcal/kg/day	Fed preferentially by EN, supplemental, or sole PN as required. Gradual increase in feeds from Day 1 to Day 4	Until ICU discharge
Shi 2019 [13]	RC, SC	30/30	81/80	67/80	NR	22/20	Ventilated patients, excepted to stay in ICU > 1 week	Measured at 3, 5, 7, 9, and 11 day	H–B formula	Aim of reaching EE, PN was 1/3 of the total, and EN was 2/3	Day 11
Zhao 2019 [15]	RC, SC	29/29	44/45	76/76	NR	19/18	Ventilated patients, excepted to stay in ICU > 1 week	Everyday	H–B formula	Fed preferentially by EN, supplemental or sole PN as required	One week
Singer 2020 [14]	RC, MC	209/208	59/61	44/53	28/28	22/22	Ventilated patients, excepted to stay in ICU > 2 days	Everyday	25 kcal/kg/day	Fed preferentially by EN, supplemental PN as required	Two weeks
Yang 2016 [23]	RC, SC	30/30	56/58	60/67	NR	14/14	Ventilated patients with sepsis	Measured at 0, 3, 7, and 14 day	H–B formula	Aim of reaching EE within 24 h, at 0, 3, 7, and 14 day (EN, PN, or EN + PN)	Two weeks

*APACHE* acute physiology and chronic health evaluation, *BMI* body mass index, *EE* energy expenditure, *EN* enteral nutrition, *H–B* Harris–Benedict, *IC* indirect calorimetry, *ICU* intensive care unit, *MC* multi-centers, *MV* mechanical ventilation, *NR* not reported, *PE* predictive equation, *PN* parenteral nutrition, *RC* randomized controlled, *SB* single blind, *SC* single-center, *SOFA* sequential organ failure assessment score

### Nutrition characteristics and delivery

Table 2 shows the nutrition characteristics and delivery in the IC and control groups. The mean measure REEs were similar between the two groups. In the IC group, a significant day-to-day variation was observed in energy targets assessed by IC. Mean energy delivered/day was described in four trials [7, 13–15, 20–22], and pooled the results showed significantly higher energy delivered in the IC group than the control group (MD = 622 kcal/day; 95% CI 407 to 837; *I*^2^ = 85%; *P* < 0.00001). Moreover, the IC group received a energy intake (89–106%) closer to the measured target than the control group (56–79%) [7, 14, 20, 21]. Mean protein delivered/day was reported in four trials [7, 14, 20, 21], and pooled the results showed a significantly higher protein delivered in the IC group when compared with the control group (MD = 20 g/day; 95% CI 16 to 25; *I*^2^ = 4%; *P* < 0.00001).

**Table 2:** Nutrition therapy

Study/year	IC device	REE targets, kcal/day, mean ± SD		Mean energy delivered/day, kcal/day, mean ± SD		Mean daily energy balance, kcal, mean ± SD		Mean protein delivered/day, g/day		Percent delivered energy over measured REE, %	
		IC	PE	IC	PE	IC	PE	IC	PE	IC	PE
Singer 2011 [7]	Deltatrac II	1976 ± 468	1838 ± 468	2086 ± 460	1480 ± 356	186 ± 206	− 312 ± 481	76 ± 16	53 ± 16	106	81
Landes 2016 [22]	Colorado Med Tech Metascope	1976 ± 481	2067 ± 341	NR	NR	87% ± 12%^b^	77% ± 17.6%^b^	NR	NR	87	77
Allingstrup 2017 [20]	COSMED Quark RMR	2069 ± 418	1887 ± 422	1877 ± 509	1061 ± 537	− 66 ± 112	− 787 ± 659.3	1.5 ± 0.4^b^	0.5 ± 0.3^b^	91	56
Gonzalez-Granda 2018 [21]	COSMED Quark RMR	21 ± 6^a^	25^a^	20 ± 6^a^	20 ± 8^a^	98% ± 8%^b^	79% ± 29%^b^	78 ± 18	59 ± 21	98	79
Shi 2019 [13]	Medgraphics, CCM	1309 ± 225	1652 ± 209	NR	NR	NR	NR	NR	NR	NR	NR
Zhao 2019 [15]	Medgraphics, CCM	1443 ± 155	1615 ± 160	NR	NR	NR	NR	NR	NR	NR	NR
Singer 2020 [14]	Deltatrac II, COVX and Quark	1953 ± 580	1942 ± 360	1746 ± 755	1301 ± 535	− 282 ± 896	− 885 ± 535	77 ± 53	62 ± 34	89	67
Yang 2016 [23]	Engestrom Carestation	1900 ± 480	1481 ± 260	NR	NR	NR	NR	NR	NR	NR	NR

*IC* indirect calorimetry, *ICU* intensive care unit, *NR* not reported, *PE* predictive equation

^a^kcal/kg/day

^b^Percent of reaching the target

^c^g/kg/day

### Clinical outcomes

**Fig. 2:**
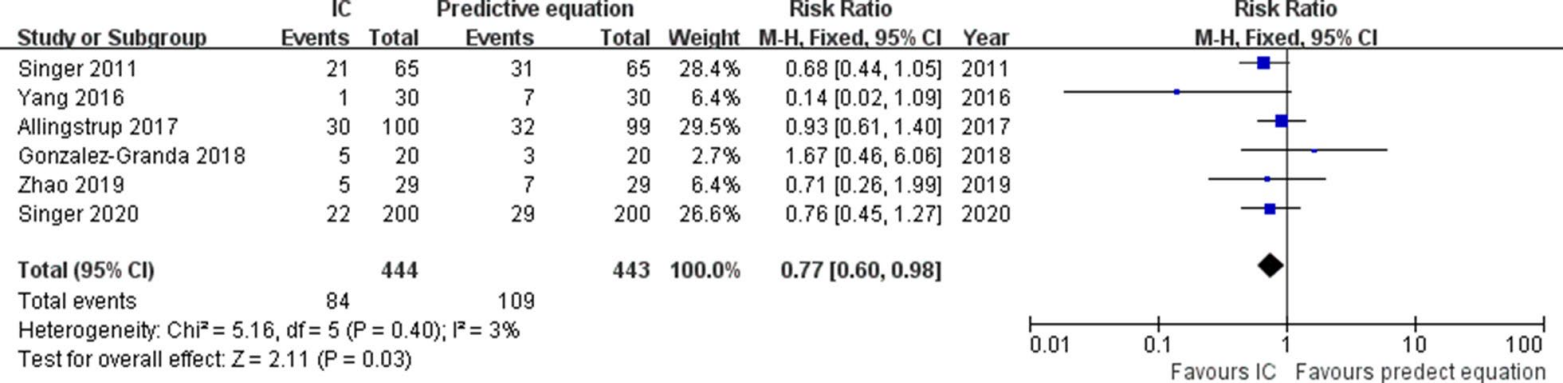
Forest plot showing the effects of energy delivery guided by indirect calorimetry on short-term mortality rate in critically ill patients

**Fig. 3:**
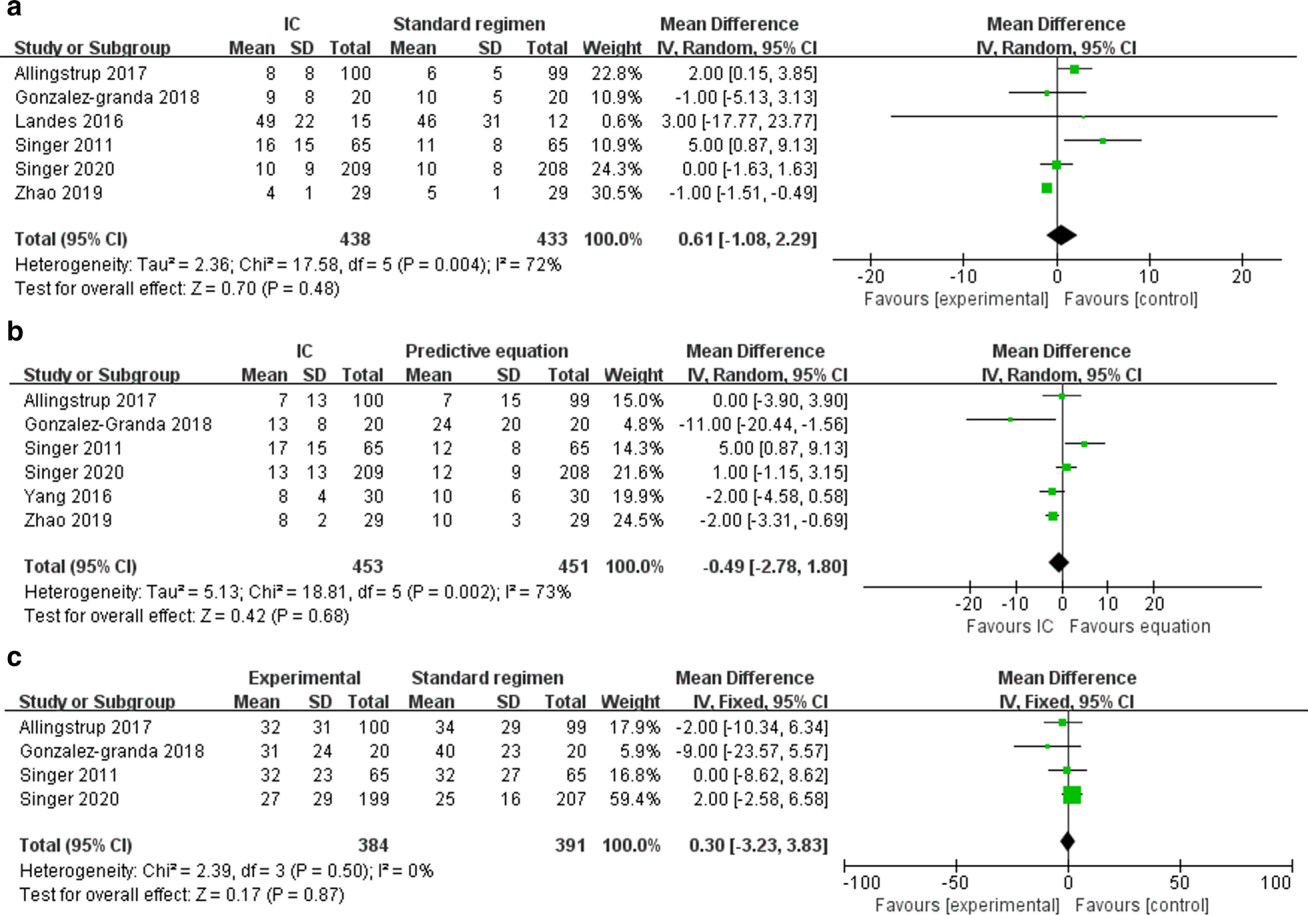
Forest plot showing the effects of energy delivery guided by indirect calorimetry on duration of mechanical ventilation (**a**), length of stay in intensive care unit (**b**) and length of stay in hospital (**c**) in critically ill patients

Short-term mortality was reported in six RCTs [7, 14, 15, 20, 21, 23]. The pooled analysis showed that compared with the control, IC improved short-term mortality (*n* = 887; RR = 0.77; 95% CI 0.60 to 0.98; *I*^2^ = 3%; *P* = 0.03) (Fig. 2). Six trials reported duration of MV as an outcome and showed no differences using IC compared to the control (*n* = 871; MD = 0.61 days; 95% CI − 1.08 to 2.29; *I*^2^ = 72%; *P* = 0.48) [7, 14, 15, 20–22] (Fig. 3a). Data from six studies found that use of IC was not associated with shorter time to ICU LOS (*n* = 904; MD = 0.32 days; 95% CI − 2.51 to 3.16; *I*^2^ = 84%; *P* = 0.82) [7, 14, 15, 20, 21, 23] (Fig. 3b). Hospital LOS was available in 4 RCTs, which was similar between IC and control groups (*n* = 775; MD = 0.30 days; 95% CI − 3.23 to 3.83; *I*^2^ = 0%; *P* = 0.87) [7, 14, 20, 21] (Fig. 3c). Only two trials reported the long-term mortality and showed similar survival between the groups [14, 20]. Infection is the most commonly reported complication, with types of infection varied. When pooled, no differences were observed between two groups in terms of all types of pneumonia (*n* = 795, RR = 1.01; 95% CI 0.58 to 1.75; *I*^2^ = 60%; *P* = 0.98) [7, 14, 20, 23], bacteremia (*n* = 329, RR = 1.1.74; 95% CI 0.90 to 3.40; *I*^2^ = 0%; *P* = 0.78) [14, 20], urinary infections (*n* = 329, RR = 1.00; 95% CI 0.49 to 9.65; *I*^2^ = 48%; *P* = 0.17) [14, 20], and abdominal infections (*n* = 329, RR = 1.03; 95% CI 0.25 to 3.90; *I*^2^ = 0%; *P* = 1.00) [14, 20]. The details in all the AEs are summarized in Additional file 5.

## Discussion

The present systematic review and meta-analysis of eight RCTs (911 patients) showed that IC-guided energy delivery significantly reduced short-term mortality in critically ill adults compared with predictive equations. The IC-guided nutrition therapy achieved higher mean energy and protein intake per day and percent delivered energy over measured REE. Additionally, using IC-guided energy delivery did not prolong the MV duration, length of stay in ICU or hospital, and increase AEs and long-term mortality.

In this updated meta-analysis, we found a better prognosis in the IC-guided group patients. This finding contrasts with a most recent meta-analysis by Tatucu-Babet et al. [11]. They reported an IC-guided energy delivery regimen showed no between-groups difference regarding mortality (both hospital and ICU mortality) and ICU LOS but a longer duration of MV. However, the authors reviewed only four trials with a total of 398 patients [7, 20–22]. By contrast, our meta-analysis had a larger sample size than the previous meta-analysis as it added four other trials published recently [12–15, 23], with more power to assess this effect. In the 2018 ESPEN guideline on ICU nutrition [10], Singer et al. performed a meta-analysis of RCTs that focused only on using IC as a calorie target and found a trend (*P* = 0.07) to improve the short-term mortality. Four RCTs were identified, of which two were included in the present meta-analysis [7, 20]. The two other trials were not included because patients recruited in the control group also received energy guided by IC measurements [9, 24].

Our findings support using IC rather than predictive equations as the gold standard to assess REE in ICU patients. Previous studies have demonstrated the low accuracy of various REE predictive equations based on weight, height, age, gender, etc., for critical illness [2, 25]. Firstly, compared with healthy individuals, REE predictive equations are frequently affected by disease characteristics such as respiratory failure, body temperature, severe trauma, burns, especially in obese or low-weight critically ill populations [8, 25]. Though adjusted by patient populations and modification factors, the estimated energy deviations still exist, and even high up to 60% [26]. Of note, researches have demonstrated that too high or too low energy supply is associated with a worse prognosis [1].

On the other hands, caloric requirements in ICU patients may change during hospitalization. These changes may be irregular and unpredicted due to the phase of critical illness, nutritional support, analgesia, neuromuscular blockade, sedation, early rehabilitation, and other unknown factors [1]. As shown in the TICACOS study [7], the authors reported a significant day-to-day variation in measured REE by IC, though the mean EE was comparable between the IC-guided and the control groups. Similar findings were also mentioned in other included studies [7, 13–15]. Therefore, an IC-guided nutrition therapy allows for capturing the daily variations of REE and matching energy supply and demand, thus avoiding the known adverse effects of under- and overfeeding due to the low precision of calculation-based REE strategy.

Our results suggested that besides accurate REE measurement, the implementation process of energy supply is also important to achieve successful nutrition therapy. In the current study, the IC group received energy intake closer to the measured targets than the control group (89–106% vs. 56–79%, respectively). This resulted in a higher cumulative caloric difference between the two groups (MD = 622 kcal/day, *P* < 0.00001). The cumulative energy deficits may harm clinical outcomes, as shown in the study by Villet et al. [27], which reported a positive correlation between the number of infection complications and cumulative energy deficits over 4 weeks of ICU stay. Another large retrospective study found that in patients receiving less than 40% of REE, the increase in energy debt harmed clinical outcomes. However, it is not the higher energy delivery, the better. Zusman et al. [1] investigated the relationship between energy delivery and REE by IC. Their results indicated a U-shaped relationship between caloric supply and mortality, with the best survival when around 70% REE was provided. This may help to explain the negative results of some included trials [7, 20]. In the TICACOS study, despite the large difference of cumulative energy balance between groups, the IC-guided group tends to overfeed, while the control group was underfed. Thus, a negative result was observed [7]. In contrast, in the EAT-ICU study [20], the median cumulative energy balance was closely related and close to and slightly below their target values (− 249 and − 747 kcal, respectively). Caloric supply in both groups was in the optimal range, and therefore, the clinical results were indistinguishable between the two groups.

On the other hands, the energy target supplied should vary according to different phases of critical illness. A previous large RCT (SPN study) measured REE by IC on day 3 to guide PN supplement and found the delivery of 100% of the energy target from days 4 with EN plus supplemental PN significantly reduced nosocomial infections [9]. Hypocaloric nutrition (less than 70% of EE) should be administered in the early phase of the illness [10]. Early full energy target feeding might lead to overfeeding and increase endogenous energy production. Moreover, the early phase of the illness is usually a period for resuscitation with frequent treatment adjustment for critically ill patients, which may hinder the accuracy of REE by IC. In the current study, some included RCTs reached 100% of the IC targets in the first 24 h after randomization [7, 21]. Again, the overfeeding during the early period of critical illness may contribute to the increased MV duration, length of ICU or hospital stay among these trials, and even infection rate, thus diluting the benefit of REE guided by IC.

Additionally, we found no differences between the groups regarding the other clinical outcomes, such as duration of MV, ICU, and hospital stay, long-term mortality, and AEs. The main explanation is that these are not the primary outcome among the most included studies. In fact, only a few or around half of the included trials have reported these outcomes. Second, for critically ill patients, ICU discharge was not always determined by the patients' condition. Third, only two trials [14, 20] had reported the long-term mortality of patients. The limited evidence suggested that IC-guided strategy did not reduce long-term mortality. Therefore, more RCTs are required to explore the effect of ET on long-term prognosis.

The current meta-analysis provides evidence to support and expands the weak suggestion in the 2018 ESPEN guidelines, i.e., using IC-guided strategy during nutrition therapy in critically ill patients. However, our meta-analysis has some limitations. First, only eight studies were included in the current review. This may be explained by the infrequent application of IC in daily clinical practice. As shown in a large prospective study, only 0.8% of the more than 8000 cases collected underwent IC measurement [28]. Moreover, we included only RCTs in the current meta-analysis to avoid the select bias of observational studies. Second, most of the included RCTs are unblinded and single-center in design and maybe likely underpowered to demonstrate actual differences in clinical and functional recovery outcomes. Third, there were differences among included trials with regards to the adopted IC measured devices, non-nutritional calories such as propofol and dextrose 5% used during the study period, enteral and parenteral formula, and patient intolerance of EN, which might result from the observed heterogeneity and thus compromise the robustness of our findings. Forth, the uneven distribution of different underlying diseases among included studies might also exert a prognostic value. For instance, one previous study showed that the higher the REE in severe sepsis, the higher the mortality. Fifth, another limitation is the non-conclusion of some randomized studies guided by IC, including two randomized trials showing a reduction of infectious complications in the patients receiving the energy determined by REE [5, 6]. This exclusion was by design as the two studies did not compare IC-guided strategy with equations. Nevertheless, these trials reinforce our conclusions. Finally, our results showed that the IC-guided group received more protein than the control group. However, we could not investigate the effect of protein delivery on ICU outcomes since it was not the study target in the current study. The timing and dose of protein delivery during critical illness remain unclear.

## Conclusion

This systemic review and meta-analysis indicate that IC-guided energy delivery significantly reduces short-term mortality in critically ill patients. Therefore, measured REE by IC should replace the conventional practice of calculation-based REE strategy.

## Supplementary Information

**Additional file 1.** Search Strategy.

**Additional file 2: Table S1.** Summarized data of predefined outcomes among the included studies.

**Additional file 3: Figure S1.** Risk-of-bias graph: review authors' judgements about each risk-of-bias item presented as percentages across all included studies. **Figure S2.** Risk of bias summary: review authors' judgements about each risk of bias item for each included study.

**Additional file 4: Figure S3.** Visual inspection of the funnel plots of included studies.

**Additional file 5: Table S2.** Complications among the included studies.

## Data Availability

All data generated or analyzed during this study are included in this published article.

## Abbreviations

CIs: Confidence intervals
EE: Energy expenditure
IC: Indirect calorimetry
ICU: Intensive care unit
IQR: Interquartile range
LOS: Length of stay
MD: Mean difference
MV: Mechanical ventilation
OR: Odds ratio
SD: Standard deviation
REE: Resting energy expenditure
RCTs: Randomized controlled trials
