# Supplementary material for: Energy delivery guided by indirect calorimetry in critically ill patients: a systematic review and meta-analysis
Source: Crit Care. 2021 Feb 27;25:88. doi: 10.1186/s13054-021-03508-6 (PMC7913168; doi:10.1186/s13054-021-03508-6)
Supplement: Supplementary file 1 — Additional file 1. Search Strategy. [file 13054_2021_3508_MOESM1_ESM.docx]

**Additional file 1 Search Strategy**

**Search Strategy** (Limited to randomized controlled trials)

Database: PubMed, Embase, Cochrane library

Search completed 25th Oct 2020

----------------------------------------------------------------------------------------------------------------------

**PubMed**

((((indirect calorimet*[Title/Abstract]) OR (((metabolic cart*[Title/Abstract]) OR (metabolic monitor*[Title/Abstract])) OR (respirat* calorimet*[Title/Abstract]))) OR (("Calorimetry, Indirect"[Mesh]) OR (((((((Calorimetries, Indirect[Title/Abstract]) OR (Indirect Calorimetries[Title/Abstract])) OR (Indirect Calorimetry[Title/Abstract])) OR (Calorimetry, Respiration[Title/Abstract])) OR (Calorimetries, Respiration[Title/Abstract])) OR (Respiration Calorimetries[Title/Abstract])) OR (Respiration Calorimetry[Title/Abstract])))) AND ((randomized controlled trial [pt] OR controlled clinical trial [pt] OR randomized [tiab] OR placebo [tiab] OR clinical trials as topic [mesh: noexp] OR randomly [tiab] OR trial [ti]) NOT (animals [mh] NOT humans [mh]))) AND (("Critical Care"[Mesh]) OR ((((critical care[Title/Abstract]) OR (critically ill[Title/Abstract])) OR (intensive care[Title/Abstract])) OR (((((((((((((((Critical Illness[Title/Abstract]) OR (Critical Care[Title/Abstract])) OR (intensive care units[Title/Abstract])) OR (Burn units[Title/Abstract])) OR (coronary care units[Title/Abstract])) OR (respiration, artificial[Title/Abstract])) ) OR (ventilators, mechanical[Title/Abstract])) OR (pulmonary ventilation[Title/Abstract])) OR (respiratory insufficiency[Title/Abstract])) OR (multiple organ failure[Title/Abstract])) OR (systemic inflammatory response syndrome[Title/Abstract])) OR (respiratory distress syndrome, adult[Title/Abstract])) OR (sepsis[Title/Abstract])) OR (shock, septic[Title/Abstract]))))

----------------------------------------------------------------------------------------------------------------------

**Embase**

#1. 'septic shock' OR 'multiple organ failure*' OR 'multiple organ dysfunction*' OR 'systemic inflammatory response' OR 'respiratory distress syndrome*' OR 'respiratory care unit*' OR 'coronary care unit*' OR 'burn unit*' OR 'high dependency unit*' OR 'intensive therapy unit' OR 'intensive treatment unit*' OR 'intensive care' OR 'critical* ill*' OR 'critical care' /mp

#2. 'lung ventilation' OR 'systemic inflammatory response syndrome' OR 'adult respiratory distress syndrome' OR 'septic shock' OR 'coronary care unit' OR 'intensive care unit' OR 'critically ill patient' OR 'intensive care'/exp

#3. #1 OR #2

#4. 'metabolic monitor*' OR 'metabolic cart*' OR 'respirat* calorimet*' OR 'indirect calorimet*'

#5. 'indirect calorimeter' OR 'indirect calorimetry'/exp

#6. #4 OR #5

#7. 'clinical trial'/exp OR 'randomization'/exp OR'single blind procedure'/exp OR 'double blind procedure'/exp OR 'randomized controlled trial'/exp OR 'crossover procedure'/exp OR 'placebo'/exp OR 'prospective studies'/exp OR ('randomi?ed controlled' NEXT/1 trial*) OR rct OR 'randomly allocated' OR 'allocated randomly' OR 'random allocation' OR (allocated NEAR/2 random) OR (single NEXT/1 blind*) OR (double NEXT/1blind*) OR ((treble OR triple) NEAR/1 blind*) OR placebo*

#8. #3 AND #6 AND #7

----------------------------------------------------------------------------------------------------------------------

**Cochrane library**

#1 MeSH descriptor: [Calorimetry, Indirect] explode all trees

#2 ("indirect calorimetry"):ti,ab,kw (Word variations have been searched)

#3 (metabolic cart):ti,ab,kw (Word variations have been searched)

#4 (respirat* calorimet*):ti,ab,kw (Word variations have been searched)

#5 #1 OR #2 OR #3 #4

#6 MeSH descriptor: [Critical Care] explode all trees

#7 MeSH descriptor: [Critical Illness] explode all trees

#8 (critically ill):ti,ab,kw (Word variations have been searched)

#9 (intensive care):ti,ab,kw (Word variations have been searched)

#10 (critical care):ti,ab,kw (Word variations have been searched)

#11 (respiratory distress syndrome):ti,ab,kw (Word variations have been searched)

#12 (sepsis):ti,ab,kw (Word variations have been searched)

#13 (shock):ti,ab,kw (Word variations have been searched)

#14 (systemic inflammatory response syndrome):ti,ab,kw (Word variations have been searched)

#15 (multiple organ failure):ti,ab,kw (Word variations have been searched)

#16 (respiratory insufficiency):ti,ab,kw (Word variations have been searched)

#17 (pulmonary ventilation):ti,ab,kw (Word variations have been searched)

#18 (burn):ti,ab,kw (Word variations have been searched)

#19 #6 OR #7 OR #8 OR #9 OR #10 OR #11 OR #12 OR #13 OR #14 OR #15 OR #16 OR #17 OR #18

#20 (randomized controlled trial):pt (Word variations have been searched)

#21 #5 AND #19 AND #20

----------------------------------------------------------------------------------------------------------------------

**Studies needed for full-reviewed but not included in the current meta-analysis (n=8 trials)**

1. Berger MM, Pantet O, Jacquelin-Ravel N, Charrière M, Schmidt S, Becce F, Audran R, Spertini F, Tappy L, Pichard C. Supplemental parenteral nutrition improves immunity with unchanged carbohydrate and protein metabolism in critically ill patients: The SPN2 randomized tracer study. Clin Nutr. 2019 Oct;38(5):2408-2416. doi: 10.1016/j.clnu.2018.10.023. Epub 2018 Nov 5.

2. Heidegger CP, Berger MM, Graf S, Zingg W, Darmon P, Costanza MC, Thibault R, Pichard C: Optimisation of energy provision with supplemental parenteral nutrition in critically ill patients: a randomised controlled clinical trial. Lancet (London, England) 2013, 381(9864):385-393.

3. Azevedo JRA, Lima HCM, Montenegro WS, Souza SCC, Nogueira I, Silva MM, Muniz NA: Optimized calorie and high protein intake versus recommended caloric-protein intake in critically ill patients: a prospective, randomized, controlled phase II clinical trial. Revista Brasileira de terapia intensiva 2019, 31(2):171-179.

4. Petros S, Horbach M, Seidel F, Weidhase L: Hypocaloric vs Normocaloric Nutrition in Critically Ill Patients: A Prospective Randomized Pilot Trial. JPEN Journal of parenteral and enteral nutrition 2016, 40(2):242-249.

5. Jonckheer J, Demol J, Lanckmans K, Malbrain MLNG, Spapen H, De Waele E. MECCIAS trial: Metabolic consequences of continuous veno-venous hemofiltration on indirect calorimetry. Clin Nutr. 2020 Apr 21:S0261-5614(20)30188-6. doi: 10.1016/j.clnu.2020.04.017. Epub ahead of print.

6. Sundström Rehal M, Liebau F, Wernerman J, Rooyackers O. Whole-body protein kinetics in critically ill patients during 50 or 100% energy provision by enteral nutrition: A randomized cross-over study. PLoS One. 2020 Oct 5;15(10):e0240045. doi: 10.1371/journal.pone.0240045.
