# Supplementary material for: Energy delivery guided by indirect calorimetry in critically ill patients: a systematic review and meta-analysis
Source: Crit Care. 2021 Feb 27;25:88. doi: 10.1186/s13054-021-03508-6 (PMC7913168; doi:10.1186/s13054-021-03508-6)
Supplement: Supplementary file 2 — Additional file 2: Table S1. Summarized data of predefined outcomes among the included studies. [file 13054_2021_3508_MOESM2_ESM.docx]

**Additional file 2**

**Table Summarized data of predefined outcomes among the included studies**

| Study/year | ICU mortality, n | | Hospital mortality, n | | Mortality 28 days, n | | Mortality 60 days, n | | Mortality 90 days, n | | Length of hospital stay, days, (mean±SD) | | Length of ICU stay, days, (mean±SD) | | Length of MV, days,  (mean±SD) | |
| --- | --- | --- | --- | --- | --- | --- | --- | --- | --- | --- | --- | --- | --- | --- | --- | --- |
|  | IC | PE | IC | PE | IC | PE | IC | PE | IC | PE | IC | PE | IC | PE | IC | PE |
| Singer 2011 ^[7]^ | 16 | 17 | 21 | 31 | NR | NR | NR | NR | NR | NR | 34±23 | 32±27 | 17±15 | 12±8 | 16±15 | 11±8 |
| Landes 2016 ^[22]^ | NR | NR | NR | NR | NR | NR | NR | NR | NR | NR | NR | NR | NR | NR | 49±22 | 46±31 |
| Allingstrup 2017 ^[20]^ | NR | NR | NR | NR | 20 | 21 | NR | NR | 30 | 32 | 30±30^b^ | 34±29^b^ | 7±13^a^ | 7±5^a^ | NR | NR |
| Gonzalez-Granda 2018 ^[21]^ | 3 | 3 | 5 | 3 | NR | NR | NR | NR | NR | NR | 31±24 | 40±23 | 13±8 | 24±20 | 9±8 | 10±5 |
| Shi 2019 ^[13]^ | NR | NR | NR | NR | NR | NR | NR | NR | NR | NR | NR | NR | NR | NR | NR | NR |
| Zhao 2019 ^[15]^ | NR | NR | NR | NR | 5 | 7 | NR | NR | NR | NR | NR | NR | 9±2 | 10±3 | 4±1 | 5±1 |
| Singer 2020 ^[14]^ | 45 | 46 | NR | NR | NR | NR | NR | NR | 22 | 29 | 27±29 | 25±16 | 13±13 | 12±9 | 10±9 | 10±8 |
| Yang 2016 ^[23]^ | NR | NR | NR | NR | 1 | 7 | NR | NR | NR | NR | NR | NR | 8±4 | 10±6 | 7±4 | 7±4 |

IC, indirect calorimetry; ICU, intensive care unit; MV, mechanical ventilation; NR, not reported; PE, predictive equation; SD,
