# Supplementary material for: Energy delivery guided by indirect calorimetry in critically ill patients: a systematic review and meta-analysis
Source: Crit Care. 2021 Feb 27;25:88. doi: 10.1186/s13054-021-03508-6 (PMC7913168; doi:10.1186/s13054-021-03508-6)

**Additional file 3**

**Figure S1. Risk of bias graph: review authors' judgements about each risk of bias item presented as percentages across all included studies.**


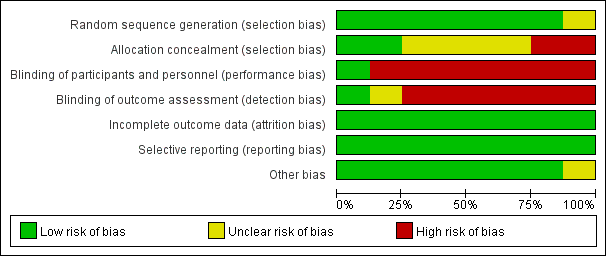


**Figure S2. Risk of bias summary: review authors' judgements about each risk of bias item for each included study.**


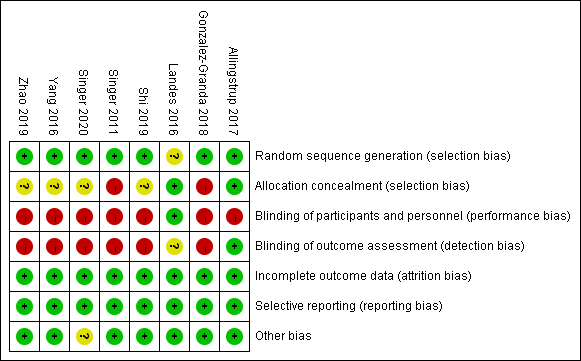

Supplement: Supplementary file 3 — Additional file 3: Figure S1. Risk-of-bias graph: review authors' judgements about each risk-of-bias item presented as percentages across all included studies. Figure S2. Risk of bias summary: review authors' judgements about each risk of bias item for each included study. [file 13054_2021_3508_MOESM3_ESM.docx]
