# Supplementary figures and images for: Energy delivery guided by indirect calorimetry in critically ill patients: a systematic review and meta-analysis
Source: Crit Care. 2021 Feb 27;25:88. doi: 10.1186/s13054-021-03508-6 (PMC7913168; doi:10.1186/s13054-021-03508-6)

**Additional file 4** **Figure S3. Funnel plot of comparison: Short-term mortality**


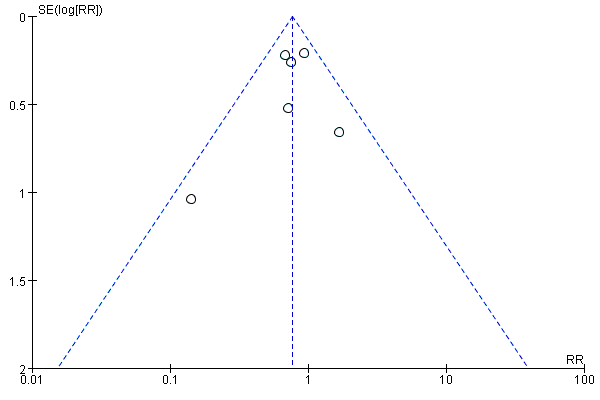

Supplement: Supplementary file 4 — Additional file 4: Figure S3. Visual inspection of the funnel plots of included studies. [file 13054_2021_3508_MOESM4_ESM.docx]
