# Supplementary material for: Energy delivery guided by indirect calorimetry in critically ill patients: a systematic review and meta-analysis
Source: Crit Care. 2021 Feb 27;25:88. doi: 10.1186/s13054-021-03508-6 (PMC7913168; doi:10.1186/s13054-021-03508-6)
Supplement: Supplementary file 5 — Additional file 5: Table S2. Complications among the included studies. [file 13054_2021_3508_MOESM5_ESM.docx]

**Additional file 5: Table S2. Complications among the included studies**

| Complications | Study/year | Sample | RR | 95% CI | P | I^2^ |
| --- | --- | --- | --- | --- | --- | --- |
| Pneumonia | Allingstrup 2017^[20]^, Singer 2011^[7]^, Singer 2020^[14]^, Yang 2016^[23]^ | 795 | 1.01 | 0.58 to1.75 | 0.98 | 60% |
| Bacteremia | Allingstrup 2017^[20]^, Singer 2011^[7]^ | 329 | 1.74 | 0.90 to 3.40 | 0.78 | 0% |
| Urinary infections | Allingstrup 2017^[20]^, Singer 2011^[7]^ | 329 | 2.19 | 0.49 to 9.65 | 0.17 | 48% |
| Abdominal infections | Allingstrup 2017^[20]^, Singer 2011^[7]^, Yang 2016^[23]^ | 389 | 1.33 | 0.69 to 2.56 | 0.87 | 0% |
| Liver impairment | Singer 2011^[7]^, Singer 2020^[14]^ | 482 | 1.00 | 0.64 to1.57 | 1.00 | 0% |
| Renal impairment | Singer 2011^[7]^, Singer 2020^[14]^ | 421 | 1.03 | 0.64 to1.57 | 0.84 | 0% |
| Unplanned surgery and surgical complications | Singer 2011^[7]^, Singer 2020^[14]^ | 463 | 1.20 | 0.79 to 1.81 | 0.88 | 0% |
| Wound infection | Singer 2011^[7]^ | 130 |  |  | 0.21 |  |
| New pressure ulcers | Singer 2011^[7]^ | 130 |  |  | 0.34 |  |
| Skin and soft-tissue infection | Allingstrup 2017^[20]^ | 199 |  |  | 0.18 |  |
| Respiratory | Singer 2020^[14]^ | 223 |  |  | 0.37 |  |
| Thoracic infection | Yang 2016^[23]^ | 60 |  |  | 0.781 |  |
| Hyperglycemia | Yang 2016^[23]^ | 60 |  |  |  |  |
| Hypoglycemia | Yang 2016^[23]^ | 60 |  |  |  |  |

RR, risk ratio; CI: confidence interval.
